# Geminin Inhibits a Late Step in the Formation of Human Pre-replicative Complexes*

**DOI:** 10.1074/jbc.M114.552935

**Published:** 2014-09-17

**Authors:** Min Wu, Wenyan Lu, Ruth E. Santos, Mark G. Frattini, Thomas J. Kelly

**Affiliations:** From the ‡ Program in Molecular Biology and; § Department of Medicine, Memorial Sloan-Kettering Cancer Center, New York, New York 10065

**Keywords:** DNA Replication, DNA-Protein Interaction, Protein Assembly, Protein Complex, Protein-Protein Interaction, Geminin, Pre-RC Assembly

## Abstract

The initial step in initiation of eukaryotic DNA replication involves the assembly of pre-replicative complexes (pre-RCs) at origins of replication during the G_1_ phase of the cell cycle. In metazoans initiation is inhibited by the regulatory factor Geminin. We have purified the human pre-RC proteins, studied their interactions *in vitro* with each other and with origin DNA, and analyzed the effects of HsGeminin on formation of DNA-protein complexes. The formation of an initial complex containing the human origin recognition complex (HsORC), HsCdt1, HsCdc6, and origin DNA is cooperative, involving all possible binary interactions among the components. Maximal association of HsMCM2–7, a component of the replicative helicase, requires HsORC, HsCdc6, HsCdt1, and ATP, and is driven by interactions of HsCdt1 and HsCdc6 with multiple HsMCM2–7 subunits. Formation of stable complexes, resistant to high salt, requires ATP hydrolysis. In the absence of HsMCM proteins, HsGeminin inhibits the association of HsCdt1 with DNA or with HsORC-HsCdc6-DNA complexes. However, HsGeminin does not inhibit recruitment of HsMCM2–7 to DNA to form complexes containing all of the pre-RC proteins. In fact, HsGeminin itself is a component of such complexes, and interacts directly with the HsMcm3 and HsMcm5 subunits of HsMCM2–7, as well as with HsCdt1. Although HsGeminin does not prevent the initial formation of DNA-protein complexes containing the pre-RC proteins, it strongly inhibits the formation of stable pre-RCs that are resistant to high salt. We suggest that bound HsGeminin prevents transition of the pre-RC to a state that is competent for initiation of DNA replication.

## Teaser

**Background:** Initiation of metazoan DNA replication is regulated by Geminin.

**Results:** HsGeminin binds to HsCdt1, HsMcm3, and HsMcm5 and inhibits formation of salt-stable complexes of purified human pre-replication proteins *in vitro*.

**Conclusion:** Geminin does not prevent association of the pre-replication proteins, but blocks a late step in pre-RC assembly.

**Significance:** This work provides mechanistic insight into the regulation of initiation of human DNA replication.

## Introduction

The initiation of eukaryotic DNA replication is a complicated and highly regulated process that involves multiple protein factors. The key features of the process have been conserved through evolution. In the first steps, the origin recognition complex (ORC)^6^ associates with origins of replication and recruits Cdc6/Cdc18 and Cdt1. The resultant complex mediates the binding of minichromosome maintenance (MCM) protein to generate the pre-replicative complex (pre-RC). The MCM protein consists of six non-identical subunits (Mcm2–7) that form the central core of the presumptive replicative helicase. Recent *in vitro* studies indicate that the pre-RC contains two MCM2–7 hexamers that surround origin DNA in a head-to-head configuration (1–3). After formation of the pre-RC, initiation of DNA replication is triggered by the activation of cyclin-dependent kinases and Dbf4-dependent kinase. These protein kinases phosphorylate key targets to promote the assembly of an active helicase containing Cdc45, Mcm2–7, and GINS (CMG complex) and the recruitment of additional proteins required for DNA synthesis (4).

ORC1–5, Cdc6, and Mcm2–7 all belong to the AAA+ family of ATPases (5, 6), and formation of the pre-RC, as well as subsequent steps in the initiation of DNA replication, requires ATP hydrolysis (7–10). Strong evidence supports a role for MCM2–7 in DNA unwinding at the replication fork. First, the highly purified heterohexameric MCM2–7 complex exhibits weak helicase activity on forked DNA structures in the presence of certain anions, such as acetate and glutamate (11). Second, a complex of MCM2–7, Cdc45, and GINS (CMG) has robust helicase activity on forked DNA substrates (12, 13). Finally, the MCM2–7 complex is absolutely required for the elongation phase of DNA replication and travels with the replisome during replication (14–16). Interestingly, a number of subcomplexes of MCM2–7 have been isolated from cell extracts, including MCM467, MCM2467, and MCM35 complexes (17–22). Of these only MCM467, which forms a hexameric complex, has been shown to have helicase activity in the absence of other factors (22–24). It is not clear whether this subcomplex plays a role in DNA replication *in vivo*.

To maintain genome integrity, the DNA replication process must occur once and only once per cell cycle. Accordingly, every step of the pre-RC assembly process is tightly regulated in all eukaryotes, although the mechanistic details vary among organisms (25). In higher eukaryotes, Geminin plays an important role in this regulation. Geminin was initially identified in *Xenopus* as an inhibitor of DNA replication that prevents the binding of MCM proteins to chromatin (26). Later studies revealed that Geminin forms a complex with Cdt1 and inhibits its interaction with MCM complexes (27–32). In addition, mouse Geminin was shown to inhibit DNA binding of Cdt1 *in vitro* (30).

Although significant progress has been achieved over the years, mainly through genetic and biochemical studies in yeast, *Drosophila*, and *Xenopus*, many of the molecular details of pre-RC assembly and its control remain unclear. In particular, the mechanism by which Geminin inhibits the initiation of DNA replication is still not well understood. Here we describe an *in vitro* system for studying human pre-RC formation utilizing exclusively purified proteins. Our results indicate that HsORC, HsCdc6, and HsCdt1 enhance the recruitment of each other, and all three proteins are required for maximal binding of HsMCM467 or HsMCM2–7 to DNA. We observed that HsGeminin inhibits the intrinsic DNA binding activity of HsCdt1, as well as the recruitment of HsCdt1 to origin DNA by HsORC and HsCdc6, but this effect is seen only in the absence of HsMCM complexes. HsCdt1 recruitment to origin DNA in complete reaction containing HsORC, HsCdc6, HsCdt1, and HsMCM467 or HsMCM2–7 is not affected by HsGeminin. HsGeminin inhibits the HsCdt1-dependent recruitment of HsMCM467 to DNA by blocking the interactions between HsCdt1 and the HsMcm6 and HsMcm7 subunits. However, HsGeminin does not significantly affect the initial recruitment of HsMCM2–7 to DNA and, in fact, is incorporated into complexes containing all of the other pre-RC components. This surprising observation is likely due to previously undescribed interactions between HsGeminin and the HsMcm3 and HsMcm5 subunits. Interestingly, HsGeminin strongly inhibits the ATP-dependent formation of stable pre-RCs that are resistant to high salt. Therefore, the inhibitory effect of HsGeminin on human pre-RC formation is likely exerted at a late stage of the pre-RC assembly process, after the recruitment of HsMCM2–7.

## EXPERIMENTAL PROCEDURES

### 

#### 

##### Antibodies

The mouse monoclonal anti-human Orc1 antibody was raised against maltose-binding protein fusion of human Orc1 fragment containing amino acids 509–860. The specificity of the antibody was confirmed by the observation that the single band corresponding to HsOrc1 in the Western blot of HeLa whole cell extract disappeared when cells were treated with siRNA of HsOrc1 (data not shown). Anti-human Orc5 polyclonal rabbit antibodies were described previously (33). Anti-FLAG M5 mouse monoclonal antibody was obtained from Sigma. Anti-c-Myc mouse monoclonal antibody (9E10), anti-human Cdc6 mouse monoclonal antibody (180.2), anti-human Mcm7 mouse monoclonal antibody (141.2), and anti-human Geminin rabbit polyclonal antibodies (FL-209) were obtained from Santa Cruz Biotechnology, Inc. Anti-human Mcm2 (4B8) and anti-human Mcm3 (3A2) mouse monoclonal antibodies were obtained from Medical & Biological Laboratories Co., LTD. Anti-human Mcm4 (A300-193A), anti-human Mcm5 (A300-195A), anti-human Mcm6 (A300-194A), and anti-human Cdt1 (A300-786A) rabbit polyclonal antibodies were obtained from Bethyl Laboratories, Inc.

##### Mutagenesis

Nucleotide changes resulting in mutations of L110A, L114A, and E116A were introduced into wild-type human Geminin coding sequence using the QuikChange site-directed mutagenesis kit (Stratagene) following the manufacturer's instructions, using MW183 (5′-AGAAAGGCGGCTTATGAAGCAGCGAAGGCAAATGAGAAAGTTCATAAA-3′)as the sense primer and MW184 (5′-TTTCTCATTTGCCTTCGCTGCTTCATAAGCCGCCTTTCTCCGTTTTTC-3′) as the antisense primer. Afterward, further mutations of N117A, E118A, H121A, and K122A were introduced into the previous QuikChange product to produce the HsGeminin BD mutant, using the sense primer MW185 (5′-GCGAAGGCAGCTGCGAAACTTGCTGCAGAAATTGAACAAAAGGACAAT-3′)and the antisense primer MW186 (5′-TTCAATTTCTGCAGCAAGTTTCGCAGCTGCCTTCGCTGCTTCATAAGC-3′).The final construct was sequenced to confirm the mutations and that no extra change was introduced.

##### Stable Cell Lines

Full-length human Mcm2 with N-terminal His_6_-2xFLAG tag was cloned into pIRESpuro3 (Clontech). The resulting plasmid was purified with a Qiagen Plasmid Maxi Kit and transfected into 293T cells using Lipofectamine 2000 reagent (Invitrogen). Transfected cells were diluted and cultured in 96-well plates at 37 °C with 5% CO_2_ in Iscove's modified Dulbecco's medium (Invitrogen) with 10% fetal bovine serum, 100 units/ml of penicillin, and 100 units/ml of streptomycin, in the presence of 5 μg/ml of puromycin for 14 days. Positive clones were further cultured, frozen, and stored in a liquid nitrogen freezer. 293T-Mcm2 clone-17 was used for large-scale purification of the HsMCM2–7 complex.

##### Protein Purifications

To purify the HsMCM2–7 complex, a clone was selected with moderate expression level of His_6_-FLAG-HsMcm2, which is high enough to enable co-precipitation of significant amounts of the other endogenous MCM subunits, whereas there would not be an excessive amount of free Mcm2 in the preparation (data not shown). Asynchronously growing 293T-Mcm2 clone-17 cells were collected by centrifugation at 100 × *g* for 10 min and washed with PBS (phosphate-buffered saline, 137 mm NaCl, 2.7 mm KCl, 4.3 mm Na_2_HPO_4_, 1.4 mm KH_2_PO_4_, pH 7.3). The harvested cells were lysed by incubation in MCMIPB (20 mm Tris-HCl, pH 7.6, 0.3 m potassium acetate, 2 mm magnesium acetate, 0.1% Tween 20, 10% glycerol) plus a protease inhibitor mixture (PIM, 2 mm PMSF, 10 μg/ml aprotinin, 10 μg/ml leupeptin, 10 μg/ml pepstatin A, and 1 mm 4-(2-aminoethyl)benzenesulfonyl fluoride) at 4 °C for 10 min, and then centrifuged at 71,000 × *g* at 4 °C for 30 min. The supernatant was incubated with protein A-agarose beads (Roche Applied Science) at 4 °C for 1 h. The supernatant was collected by centrifugation at 800 × *g* at 4 °C for 3 min, and then incubated with anti-FLAG M2-agarose beads (Sigma) at 4 °C for 3 h. The resin was washed once with MCMIPB, and then twice with MCMB (20 mm Tris-HCl, pH 7.6, 0.3 m potassium acetate, 2 mm magnesium acetate, 0.01% Tween 20, 10% glycerol), each time for 10 min at 4 °C. The HsMCM2–7 complex was eluted with MCMB plus 4 mg/ml of FLAG peptide (Sigma) at 4 °C overnight. A typical purification yielded 20 μg of purified protein out of a culture of 140 × 10^6^ cells.

The coding sequences of N-terminal Myc-tagged human Cdc6, N-terminal FLAG-tagged human Cdt1, N-terminal FLAG-tagged and C-terminal His_6_-tagged wild-type and BD mutant human Geminin, N-terminal His_6_-FLAG-tagged human Mcm4, and untagged human Mcm2, Mcm3, Mcm4, Mcm5, Mcm6, and Mcm7 were amplified by PCR and subcloned into pFastBac1 (Invitrogen), and the baculoviruses were made using the Bac-to-Bac system following the manufacturer's instructions (Invitrogen). Sf9 insect cells were cultured at 27 °C in Grace's medium (Invitrogen) with 10% fetal bovine serum, 100 units/ml of penicillin, and 100 units/ml of streptomycin. For expression of recombinant proteins, Sf9 cells were infected with the recombinant baculoviruses at a multiplicity of infection of 5, and cultured for 48 h. The cells were collected by centrifugation at 800 × *g* for 10 min and washed with PBS.

To purify Myc-HsCdc6, Sf9 cells expressing Myc-HsCdc6 were lysed by incubation in IPB (PBS plus 5 mm EDTA, 5 mm EGTA, 1 mm Na_3_VO_4_, 10 mm Na_4_P_2_O_7_, 50 mm NaF, pH 7.4, 1% Triton X-100, 0.5 m NaCl) plus PIM at 4 °C for 5 min, and then centrifuged at 71,000 × *g* at 4 °C for 30 min. The supernatant was incubated with anti-c-Myc (9E10) antibody-conjugated agarose beads (Santa Cruz Biotechnology, Inc.) at 4 °C for 3 h. The resin was washed 3 times with PBS plus 0.5 m NaCl with a 10-min incubation at 4 °C each time. The bound protein was eluted with PSB (20 mm Tris-HCl, pH 7.6, 10% glycerol, 0.1 m NaCl, 0.1 mm dithiothreitol, 1 mm EDTA, 0.05% Triton X-100) plus 2 mg/ml of 9E10 peptide (Covance) overnight at 4 °C. A typical purification yielded 120 μg of purified protein out of a culture of 150 × 10^6^ cells.

To purify FLAG-HsCdt1, Sf9 cells expressing FLAG-HsCdt1 were lysed in ISLB (10 mm Tris-HCl, pH 6.8, 0.4 m sorbitol, 150 mm potassium acetate, 5 mm MgCl_2_, 5 mm MgSO_4_, and 1% Triton X-100) plus PIM and 0.5 m NaCl at 4 °C for 5 min, and then centrifuged at 71,000 × *g* at 4 °C for 30 min. The supernatant was incubated with anti-FLAG M2-agarose beads (Sigma) at 4 °C for 3 h. The beads were washed 3 times with PBS plus 0.5 m NaCl and 1% Triton X-100 with a 10-min incubation at 4 °C each time. The bound protein was eluted with PSB plus 0.4 mg/ml of FLAG peptide. A typical purification yielded 240 μg of purified protein out of a culture of 20 × 10^6^ cells.

To purify wild-type or BD mutant FLAG-HsGeminin-His_6_, Sf9 cells expressing corresponding proteins were lysed in NLB (50 mm NaH_2_PO_4_, 0.5 m NaCl, 1% Triton X-100, pH 8.0) plus 10 mm imidazole and PIM at 4 °C for 10 min, and then centrifuged at 71,000 × *g* at 4 °C for 30 min. The supernatant was incubated with Ni-NTA beads (Qiagen) at 4 °C for 2 h. The resin was washed 3 times with NLB plus 20 mm imidazole, with a 10-min incubation at 4 °C each time. The bound proteins were eluted twice with NLB plus 0.25 m imidazole, with incubation at 4 °C for 30 min each time. The eluates were combined and incubated with anti-FLAG M2-agarose beads at 4 °C for 3 h. The beads were washed 3 times with PBS plus 0.5 m NaCl and 1% Triton X-100, and once with PSB, with a 10-min incubation at 4 °C each time. The bound proteins were then eluted with PSB plus 1 mg/ml of FLAG peptide. A typical purification yielded 48 μg of purified protein out of a culture of 30 × 10^6^ cells.

To purify the HsMCM467 complex, Sf9 cells were infected with viruses expressing His_6_-2xFLAG-HsMcm4, HsMcm6, and HsMcm7. After 48 h, cells were lysed in MCMIPB, and then centrifuged at 71,000 × *g* at 4 °C for 30 min. The supernatant was incubated with Ni-NTA beads at 4 °C for 2 h, and then the resin was washed 5 times with MCMIPB with 10 mm imidazole, followed by three elutions with MCMIPB with 0.25 m imidazole. The pooled eluates were incubated with FLAG M2-agarose beads overnight at 4 °C. The beads were washed 5 times with MCMIPB, followed by two elutions with MCMIPB with 2 mg/ml of FLAG peptide at 4 °C. The eluted proteins were dialyzed against M467B (25 mm Hepes, pH 7.6, 2 mm magnesium acetate, 50 mm potassium glutamate, 0.5 mm DTT, 0.05% Tween 20, and 10% glycerol) at 4 °C. A typical purification yielded 300 μg of purified protein out of a culture of 400 × 10^6^ cells.

All purified proteins were analyzed with the silver staining procedure and the identities of the proteins were confirmed by mass spectrometric analysis (Microchemistry & Proteomics Core Facility, MSKCC). The concentrations of the protein preparations were estimated by comparing the intensities of individual protein bands on silver-stained polyacrylamide gels to those of the Perfect Protein Markers (Novagen).

##### DNA-coupled Magnetic Beads

A 2-kb fragment (lamin B2) corresponding to positions 2500–4500 bp of the human *lamin B2* origin (GenBank^TM^ accession number M94363) was synthesized by PCR using the sense primer MW162 (5′-CGGGATCCTGCAGCTCAAGTCTTAAAGAC-3′), and the antisense primer MW163 (5′-GGGGTACCGGACTACAACTCCCACACGAC-3′), and subcloned into pUC19 (New England BioLabs).

The pUC19-lamin-B2 plasmids were biotinylated using PHOTOPROBE long-arm biotin (Vector Laboratories) according to the manufacturer's instruction. To generate biotinylated linear lamin B2 DNA, the 2-kb fragment was amplified by PCR with the primer MW162 labeled at the 5′ end with biotin. The PCR product was subjected to agarose gel electrophoresis, after which the corresponding band was excised and the lamin B2 fragment was purified with QiaQuick gel extraction kit (Qiagen) following the manufacturer's instruction.

The immobilization of biotinylated DNA to magnetic beads was carried out using the Dynabeads kilobaseBINDER kit (Invitrogen) according to the manufacturer's instruction. The amount of DNA bound to the beads was estimated by subtracting the amount of DNA in the flow-through after binding from the amount that was put in the reaction. Control beads without DNA underwent the same coupling procedure in the absence of DNA.

##### In Vitro Pre-RC Binding

In a typical binding reaction, there were ∼16 nm linear lamin B2 DNA molecules or 6 nm pUC19-lamin-B2 molecules. HsORC, HsCdc6, HsCdt1, HsMCM467, and HsMCM2–7 were used at the concentration of 13 nm, and HsGeminin at 200 nm unless indicated otherwise. In Fig. 4*B*, 26 nm HsMCM467, 13 nm HsMCM2–7, and 60 nm HsGeminin were used. When HsGeminin was used, it was preincubated with HsCdt1 at 4 °C for 30 min, unless indicated otherwise. The reactions were carried out in Thermomixer R (Eppendorf) under constant agitation at 1,100 rpm, and the beads were collected with the 3 in 1 MPS magnetic particle separator (PureBiotech, LLC).

In the absence of HsMCM complexes, the reactions were carried out in one step. Proteins were incubated with DNA beads in GSB (50 mm Hepes, pH 7.5, 5 mm MgCl_2_, 0.1 mm EDTA, 0.1 mm EGTA, 1 mm dithiothreitol, 0.1% Tween 20, 0.1 m NaCl, 0.12 mg/ml bovine serum albumin (BSA), and 1 mm ATP) at 37 °C for 30 min. The beads were then washed twice with MWB (30 mm Hepes, pH 7.6, 7 mm magnesium acetate, 1 mm dithiothreitol, 1 mm 4-(2-aminoethyl)benzenesulfonyl fluoride, 0.25 m sucrose, 100 mm potassium glutamate, and 0.05% Nonidet P-40).

When HsMCM complexes were used, the reactions were performed in two steps. HsORC was first incubated with DNA beads in GSB at 37 °C for 30 min. The beads were washed twice with MWB, and then incubated with other proteins in MCMBB (20 mm Hepes, pH 7.6, 6 mm Tris-HCl, 90 mm potassium acetate, 7.6 mm magnesium acetate, 1 mm dithiothreitol, 1 mm 4-(2-aminoethyl)benzenesulfonyl fluoride, 0.25 m sucrose, 0.1 mg/ml BSA, 0.03% Tween 20, 3% glycerol, and 4 mm ATP) at 37 °C for 30 min. Afterward, the beads were washed twice with MWB at 4 °C with 1 min of agitation at 1,100 rpm each time. To isolate the salt-resistant HsMCM complexes, the beads were subjected to one additional wash in MWB plus 0.3 or 0.5 m NaCl in the absence of potassium glutamate at 4 °C with 5 min of agitation at 1,100 rpm.

To analyze the proteins bound to DNA beads, the beads were incubated with 2× SDS SB (100 mm Tris-HCl, pH 6.8, 200 mm dithiothreitol, 4% SDS, 0.2% bromphenol blue, and 20% glycerol) at 100 °C for 10 min, and then centrifuged at 16,100 × *g* for 1 min. After SDS-PAGE of the supernatant, the proteins were transferred to a nitrocellulose membrane and Western blotting with appropriate antibodies was performed. Quantifications of band densities were performed with a GS-900 Calibrated Densitometer (Bio-Rad) according to manufacturer's instruction. Standard deviation values were calculated based on 3 or more independent experiments.

##### Immunoprecipitations

To investigate the interactions between HsMCM subunits and HsCdc6, HsCdt1, or HsGeminin, Sf9 insect cells expressing each individual protein were lysed in PBS plus 1% Triton X-100 and PIM, and the suspension was centrifuged at 60,000 × *g* at 4 °C for 30 min. The supernatant from cells expressing each HsMCM subunit was combined with that of cells expressing Myc-HsCdc6, FLAG-HsCdt1, or FLAG-HsGeminin-His_6_ or control extract of uninfected Sf9 cells, and was then incubated with epitope-specific agarose beads at 4 °C for 3 h. Afterward, the beads were washed three times with PBS.

To study interactions between HsCdt1 and wild-type or BD mutant HsGeminin, 60 ng of purified FLAG-HsCdt1 was incubated with 40 ng of wild-type or mutant HsGeminin, or control buffer in the presence of 10 μl of protein A/G plus agarose suspension (Santa Cruz Biotechnology, Inc.) and 60 ng of rabbit anti-human Geminin polyclonal antibodies in PBS plus 1 mg/ml BSA and PIM at 4 °C for 3 h. The beads were then washed three times with PBS plus 0.5 m NaCl.

To analyze the precipitated proteins, agarose beads were resuspended in 2× SDS SB, incubated at 100 °C for 10 min, and centrifuged at 16,100 × *g* for 1 min. After SDS-PAGE of the supernatant, the proteins were transferred to a nitrocellulose membrane and Western blotting analyses with appropriate antibodies were performed.

## RESULTS

**FIGURE 1.**
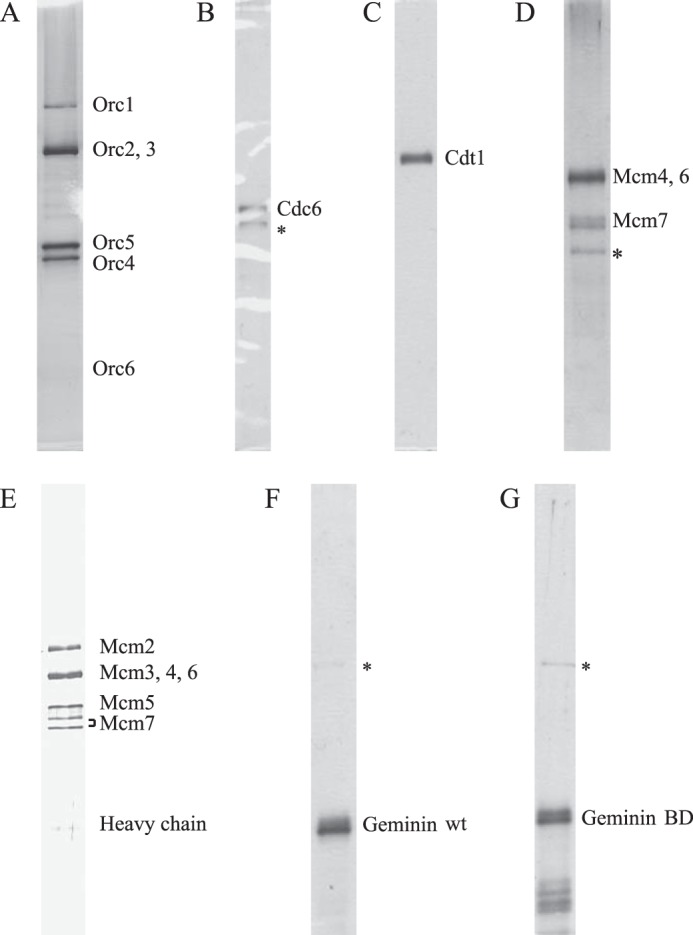
**Analysis of purified human pre-RC components by SDS-PAGE followed by silver staining.** *A*, HsORC. *B*, Myc-HsCdc6 (the faster migrating band (*) is a degradation product of HsCdc6 as demonstrated by analysis of tryptic peptides by mass spectrometry). *C*, FLAG-HsCdt1. *D*, HsMCM467 (the *asterisk* indicates the 70-kDa insect heat shock protein). *E*, HsMCM2–7. *F*, wild-type HsGeminin (the *asterisk* indicates the 70-kDa insect heat shock protein). *G*, mutant HsGeminin deficient in Cdt1 binding (HsGeminin-BD) (the *asterisk* indicates the 70-kDa insect heat shock protein).

Previous studies in the *Xenopus* system have shown that Geminin inhibits pre-RC assembly, and therefore prevents *de novo* formation of the pre-RC during S, G_2_, and M phases, as one of the mechanisms that eukaryotic cells employ to prevent re-replication (26). To study the effect of HsGeminin on human pre-RC formation *in vitro*, we purified all known protein components involved in this process, and studied the requirements for pre-RC assembly on DNA coupled to magnetic beads. The purified proteins were free of significant contamination as assessed by SDS-polyacrylamide gel electrophoresis (Fig. 1). Recombinant human ORC was purified by affinity chromatography from Sf9 insect cells co-infected with baculoviruses encoding the six HsOrc subunits, as previously described (34). Consistent with our previous observations, the HsOrc1 and HsOrc6 subunits were substoichiometric in the purified complex (Fig. 1*A*). HsCdc6, tagged at the N terminus with Myc epitope (Fig. 1*B*), and HsCdt1, tagged at the N terminus with FLAG epitope (Fig. 1*C*), were also expressed in Sf9 cells and purified to near homogeneity by affinity chromatography. Recombinant human Mcm4 (tagged at the N terminus with His_6_ and FLAG epitopes), Mcm6, and Mcm7 were co-expressed in Sf9 cells, and HsMCM467 complexes were purified by affinity chromatography (Fig. 1*D*). Recombinant wild-type HsGeminin (Fig. 1*F*), and a mutant form of HsGeminin that is unable to interact with HsCdt1 (Fig. 1*G*, described later), both tagged at the N terminus with the FLAG epitope and at the C terminus with His_6_, were expressed in Sf9 cells, and purified by two steps of affinity chromatography, using Ni-NTA beads followed by FLAG antibody-conjugated agarose beads.

For reasons that are unclear, we were unable to obtain significant quantities of HsMCM2–7 following co-infection of Sf9 cells with recombinant baculoviruses encoding the six subunits. Therefore, we generated 293T cell lines stably expressing HsMcm2 tagged with the His_6_ and FLAG epitopes. The HsMCM2–7 complex was purified to near homogeneity from asynchronously growing cells using anti-FLAG antibody-conjugated agarose beads (Fig. 1*E*). The presence of each MCM subunit in the preparation was confirmed by mass spectrometry. It has been reported that *S. cerevisiae* Cdt1 co-purifies with MCM2–7 (2, 35). However, no HsCdt1 was detected in our HsMCM2–7 preparations, either by mass spectrometry or Western blotting analysis (data not shown). Immunoprecipitation of purified HsMCM2–7 with anti-HsMcm3 monoclonal antibody revealed that a significant amount of HsMcm2, and almost all of HsMcm4, -5, -6, and -7 co-precipitated with HsMcm3 (data not shown). Therefore, the majority of MCM complexes in the preparation contained all six MCM subunits.

### 

#### 

##### In the Absence of MCM Complexes HsGeminin Inhibits the Recruitment of HsCdt1 to Origin DNA

As the first step toward defining the requirements for assembly of the human pre-RC, we studied the interactions of highly purified human ORC, Cdc6, and Cdt1, the homologues of which are known to be required for loading the MCM complexes onto DNA in other systems.

**FIGURE 2.**
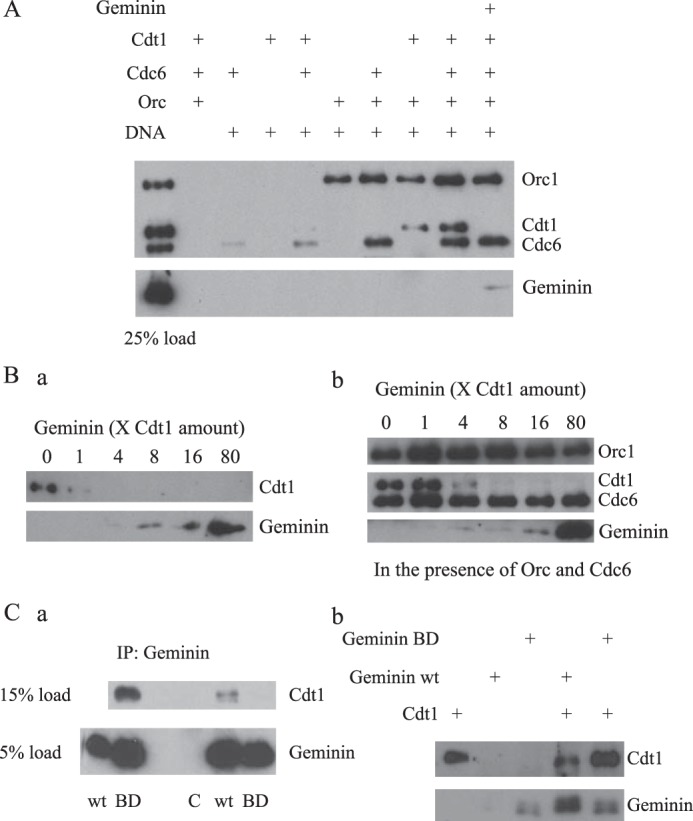
**Interactions of purified recombinant human ORC, Cdc6, and Cdt1 with DNA, and inhibition of the DNA-binding activity of HsCdt1 by HsGeminin.** *A*, DNA-binding assays. Control magnetic beads or beads with plasmid DNA containing the *lamin B2* origin of DNA replication were incubated with the indicated proteins in a two-step reaction (see ”Experimental Procedures“ for details). The bound proteins were detected by Western blotting. *B*, HsGeminin inhibits DNA binding by HsCdt1. HsCdt1 was incubated with control buffer or the indicated amount of HsGeminin, and then the protein mixture was incubated with magnetic beads containing pUC19-lamin-B2 plasmid DNA in the absence (*a*) or presence (*b*) of HsORC and HsCdc6. *C*, interaction between HsCdt1 and HsGeminin is necessary for inhibition of HsCdt1 DNA binding activity. *a*, co-immunoprecipitation (*IP*) of HsCdt1-HsGeminin complexes. Purified HsCdt1 was incubated with control buffer (*C*), purified wild-type HsGeminin (*wt*) or purified HsGeminin BD mutant (*BD*), and complexes were collected on protein A/G plus-agarose beads containing anti-HsGeminin polyclonal antibodies. *b*, DNA-binding assay. HsCdt1 was preincubated with wild-type (*wt*) or BD mutant (*BD*) HsGeminin, or control buffer, and then incubated with magnetic beads containing linear lamin B2 DNA fragments.

DNA binding assays were performed with circular plasmid DNA containing a 2-kb fragment from the human *lamin B2* origin of DNA replication (36). The DNA was coupled to magnetic beads and incubated with proteins in a buffer containing 0.1 m NaCl and 1 mm ATP at 37 °C for 30 min. DNA-bound proteins were analyzed by SDS-PAGE and Western blotting. As shown in Fig. 2*A*, none of the purified proteins showed detectable binding to control beads without DNA. As expected, HsORC efficiently associated with DNA-coupled beads (>25% of input) in the absence of the other factors, and the recruitment of HsORC was enhanced by the presence of HsCdc6. Both HsCdc6 and HsCdt1 were also capable of binding to DNA beads in the absence of other factors, albeit with relatively low efficiency. Interestingly, the association of both proteins increased modestly when they were incubated together, suggesting that HsCdc6 and HsCdt1 can form a complex on DNA in the absence of HsORC. The binding of both HsCdc6 and HsCdt1 was also enhanced significantly in the presence of HsORC, and maximal association was achieved when all three proteins were present (Fig. 2*A*). Similar results were obtained when the beads contained only the linear 2-kb fragment of the *lamin B2* origin DNA (data not shown), consistent with our previous observation that the binding of HsORC to DNA is sequence-independent (34). These observations indicate that the formation of the ternary complex containing HsORC, HsCdc6, and HsCdt1 is cooperative with all three proteins interacting with each other and with origin DNA.

Previous work in several vertebrate systems has shown that Geminin interacts with Cdt1 and inhibits the loading of the MCM complex at origins of DNA replication, but the mechanism of inhibition is poorly understood. Preincubation with ∼16-fold excess of HsGeminin at 4 °C for 30 min greatly reduced the binding of HsCdt1 to origin DNA in the presence of HsORC and HsCdc6 (Fig. 2*A*). Titration experiments with varying amounts of HsGeminin revealed that HsGeminin was able to significantly reduce the binding of isolated HsCdt1 to DNA when the HsGeminin:HsCdt1 ratio was 1:1, consistent with previous studies of mouse Cdt1 (30) (Fig. 2*B, a*). HsGeminin also completely inhibited the recruitment of HsCdt1 to DNA in the presence of HsORC and HsCdc6, but somewhat higher concentrations of HsGeminin were required, presumably because of the additional interactions of HsCdt1 with other pre-RC proteins (Fig. 2*B, b*). Studies in the *Xenopus* system have shown that the concentration of endogenous Cdt1 in egg extracts is 25–30 nm, and the concentration of Geminin is 50 nm. In nucleoplasmic extracts, where Geminin exhibits its inhibitory effects, Cdt1 is not detectable, and the concentration of Geminin is in the 160–1000 nm range (37, 38). Therefore, the concentrations of HsCdt1 (13 nm) and HsGeminin (200 nm) used in this study are similar to the physiological levels of these proteins.

In control experiments, we verified that interaction between HsCdt1 and HsGeminin is required for the inhibitory effect of HsGeminin on the DNA binding activity of HsCdt1. Based on the crystal structure of the mouse Geminin-Cdt1 complex, we generated a mutant form of HsGeminin, with multiple alanine substitutions (L110A/L114A/E116A/N117A/E118A/H121A/K122A) in the domain required for binding to Cdt1 (31). Immunoprecipitation experiments with polyclonal antibodies against HsGeminin demonstrated that purified recombinant HsCdt1 and wild-type HsGeminin form a complex that is stable in 0.5 m NaCl, whereas the mutant protein, HsGeminin-BD, failed to interact with HsCdt1 under the same conditions (Fig. 2*C, a*). HsGeminin-BD did not inhibit the binding of HsCdt1 to DNA (Fig. 2*C, b*).

Interestingly, the binding of HsGeminin to DNA was enhanced in the presence of HsCdt1, whereas HsGeminin alone did not bind DNA (Fig. 2*C, b*). Thus, it appears that HsCdt1-HsGeminin complexes have some residual affinity for DNA, albeit much less than the affinity of free HsCdt1. In agreement with this finding, we observed that purified HsCdt1-HsGeminin complexes were able to bind to DNA (data not shown). This finding is consistent with previous observations suggesting that mouse Geminin can form ternary complexes with Cdt1 and DNA (30, 39). HsCdt1 did not enhance the recruitment of HsGeminin-BD to DNA (Fig. 2*C, b*). Therefore, interaction between HsCdt1 and HsGeminin is necessary for both the inhibition of the DNA-binding activity of HsCdt1 and the formation of ternary HsCdt1-HsGeminin-DNA complexes.

##### Differential Effects of HsGeminin on the Recruitment of HsMCM467 and HsMCM2–7 Complexes to DNA

A crucial step in the initiation of DNA replication is the recruitment of the MCM helicase to the replication origin. ORC, Cdc6, and Cdt1 have all been shown to play important roles in this step in a number of model organisms. Using purified proteins, we studied the requirements for the recruitment of HsMCM2–7, the complete heterohexameric human MCM complex, as well as HsMCM467, a hexameric subcomplex of MCM that exhibits weak helicase activity (22–24).

**FIGURE 3.**
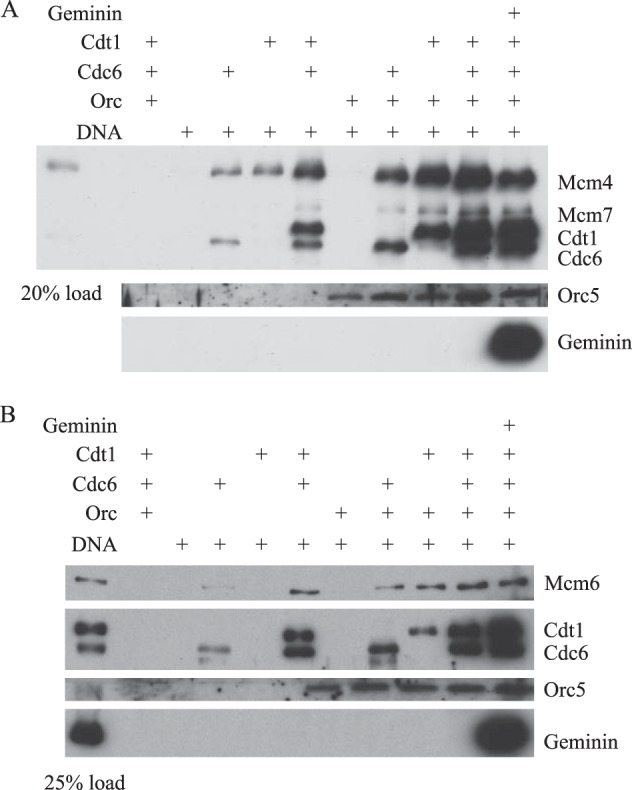
**Requirements for recruitment of purified HsMCM complexes to DNA.** *A*, requirements for HsMCM467 recruitment to origin DNA. Magnetic beads containing pUC19-lamin-B2 plasmid DNA were incubated with HsORC or control buffer for 30 min, and then further incubated with HsMCM467 in the presence of HsCdc6 and/or HsCdt1 for 30 min. HsCdt1 was preincubated with control buffer or HsGeminin as indicated. *B*, requirements for recruitment of HsMCM2–7 to origin DNA. Reaction mixtures were assembled and incubated as described in *A*, except that HsMCM2–7 was used in place of HsMCM467.

As shown in Fig. 3*A*, the purified HsMCM467 complex did not bind to DNA in the absence of the other factors. The presence of HsORC was insufficient to recruit HsMCM467 to DNA, but in the presence of either HsCdc6 or HsCdt1 recruitment of a small fraction of the added HsMCM467 was observed. Interestingly, substantial binding of HsMCM467 (>50% of input) was observed when HsCdc6 and HsCdt1 were both present, but HsORC was absent. This observation confirms that a complex of HsCdt1 and HsCdc6 has DNA-binding activity and further demonstrates that the complex is capable of recruiting HsMCM467. The presence of HsORC enhanced the recruitment of both HsCdc6 and HsCdt1 to DNA and resulted in maximal binding of HsMCM467. Taken together, these observations suggest that the recruitment of HsMCM467 to origin DNA depends largely upon direct interactions with the pre-RC proteins, HsCdc6 and HsCdt1. Our data are consistent with the possibility that HsORC plays only an indirect role in HsMCM467 recruitment via stabilization of the binding of an HsCdc6-HsCdt1 complex. Alternatively, the binding of HsCdc6 and HsCdt1 may alter the conformation of HsORC, exposing sites for direct HsORC-HsMCM interactions.

**FIGURE 4.**
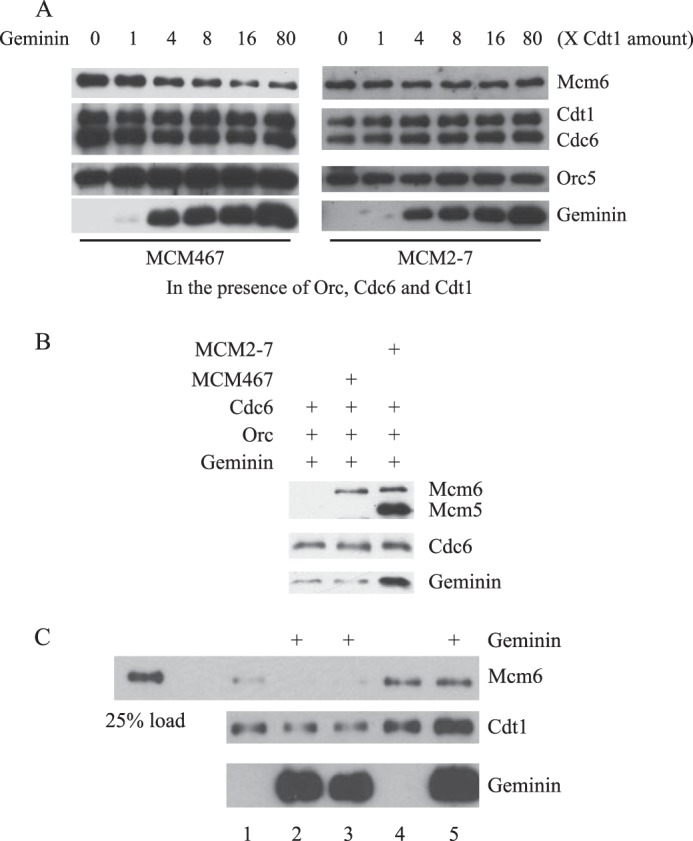
**Differential effects of HsGeminin on the recruitment of HsMCM467 and HsMCM2–7 complexes to DNA.** *A*, comparison of the effects of HsGeminin on recruitment of HsMCM2–7 and HsMCM467. Magnetic beads containing pUC19-lamin-B2 plasmid DNA were incubated with HsORC for 30 min, and then further incubated with HsCdc6 and HsCdt1 in the presence of HsMCM2–7 or HsMCM467 for 30 min. HsCdt1 was preincubated with control buffer or the indicated amount of HsGeminin. *B*, binding of HsGeminin to HsMCM2–7. Magnetic beads containing lamin B2 DNA fragments were incubated with HsORC and HsCdc6 for 30 min, and then further incubated with either HsMCM467 or HsMCM2–7 for 30 min, followed by incubation with HsGeminin for 30 min. *C*, recruitment of HsMCM467 to origin DNA is not reversed by HsGeminin. Magnetic beads containing pUC19-lamin-B2 plasmid DNA were preincubated with HsORC for 30 min, and then further incubated with HsCdt1 and HsMCM467 for 20 (*lanes 1–3*) or 40 min (*lanes 4* and *5*), in the presence of either control buffer or HsGeminin. HsGeminin or control buffer was added at various times as follows: *lane 1*: control buffer was added at *t* = −30 min (preincubated with HsCdt1 for 30 min prior to addition to the reaction). *Lane 2*, HsGeminin was added at *t* = −30 min. *Lane 3*, HsGeminin was added at *t* = 0 min (the start of second incubation). *Lane 4*, control buffer was added at t = +20 min (20 min after the second incubation started). *Lane 5*, HsGeminin was added at *t* = +20 min.

Preincubation of HsCdt1 with ∼16-fold excess of HsGeminin resulted in a modest reduction in the recruitment of HsMCM467 to a level slightly greater than that observed when HsCdt1 was not included in the reaction mixture (27 ± 10% of the level of binding without HsGeminin) (Fig. 3*A*). More detailed analysis revealed that a relatively high concentration of HsGeminin, 4–8-fold higher than that of HsCdt1, was required to reduce the binding of HsMCM467 by 50% and that significant binding was detectable even at an HsGeminin concentration 80-fold higher than that of HsCdt1 (Fig. 4*A*). The observed inhibition of HsMCM467 binding was correlated with increased binding of HsGeminin. We also observed that the binding of HsCdt1 was considerably more resistant to inhibition by HsGeminin in the presence than in the absence of HsMCM467 (compare the titration shown in Figs. 2*B* and 4*A*). The greater stability of HsCdt1 is presumably due to the mutual interactions of HsCdt1, HsCdc6, and HsMCM467 noted above. Thus, our data indicate that DNA-protein complexes containing both HsCdt1 and HsGeminin can form at high HsGeminin concentrations, but these complexes are less competent for recruitment of HsMCM467 than complexes formed in the absence of HsGeminin.

Although it is clear that preincubation of HsGeminin with HsCdt1 is sufficient to reduce recruitment of HsMCM467, we were also interested in the question of whether HsGeminin could reverse the recruitment of HsMCM467 to origin DNA once it had occurred. To assess this possibility, we studied the effects of varying the time of HsGeminin and HsCdt1 addition to reaction mixtures containing HsORC-bound DNA (Fig. 4*C*). We observed that HsGeminin inhibited the HsCdt1-dependent recruitment of HsMCM467, when it was preincubated with HsCdt1 or when it was added to HsORC-bound DNA simultaneously with HsCdt1 (Fig. 4*C*, *lanes 2* and *3*). However, the addition of HsGeminin to HsORC-bound DNA 20 min after the addition of HsCdt1 had no effect on the total amount of HsMCM467 recruited (Fig. 4*C*, compare *lanes 4* and *5*). This result demonstrates that complexes of HsMCM467 with HsORC, HsCdt1, and HsCdc6 are quite stable and do not appreciably dissociate in the presence of HsGeminin.

The requirements for recruitment of the complete heterohexameric HsMCM2–7 complex to DNA were similar to those observed for recruitment of HsMCM467 (Fig. 3*B*). HsMCM2–7 alone did not bind to plasmid DNA and exhibited only weak binding in the presence of HsORC or HsORC plus HsCdt1 or HsCdc6. Maximal recruitment of HsMCM2–7 was obtained when HsORC, HsCdc6, and HsCdt1 were all present. Surprisingly, preincubation of HsCdt1 with ∼16-fold excess of HsGeminin did not inhibit the recruitment of HsMCM2–7 to DNA, in contrast to the result obtained with HsMCM467. Fig. 4*A* shows a direct comparison of the effects of HsGeminin on recruitment of HsMCM467 and HsMCM2–7 by HsORC, HsCdc6, and HsCdt1. Although preincubation of HsGeminin with HsCdt1 reduced the recruitment of HsMCM467 as described above, the same treatment had no effect on HsMCM2–7 association even at a concentration of HsGeminin 80-fold greater than that of HsCdt1. Again, we observed that the complexes formed on origin DNA contained HsCdt1 and HsGeminin, as well as HsMCM2–7. We conclude from these experiments that HsGeminin does not inhibit the initial association of HsMCM2–7 with the other pre-RC factors at origins of DNA replication. In fact, HsGeminin is a component of complexes containing all of the pre-RC proteins. The explanation for this finding is presented below.

##### HsGeminin Interacts with HsMCM2–7 Complex

The finding that HsGeminin does not inhibit the recruitment of HsMCM2–7 was unexpected, given previous studies in *Xenopus* egg extracts suggesting that interaction of Geminin with Cdt1 blocks binding of MCM complexes at origins of DNA replication and given our observation that the binding of HsCdt1 to DNA is strongly inhibited by preincubation with HsGeminin (Fig. 2). Among the potential explanations for this finding, we considered the possibility that HsGeminin might interact with HsMCM2–7 and stabilize its binding. This possibility was supported by the observation that HsORC plus HsCdc6 can recruit both HsMCM2–7 and HsMCM467 to DNA, but significant association of the resulting complexes with HsGeminin is only observed in the presence of HsMCM2–7 (Fig. 4*B*).

**FIGURE 5.**
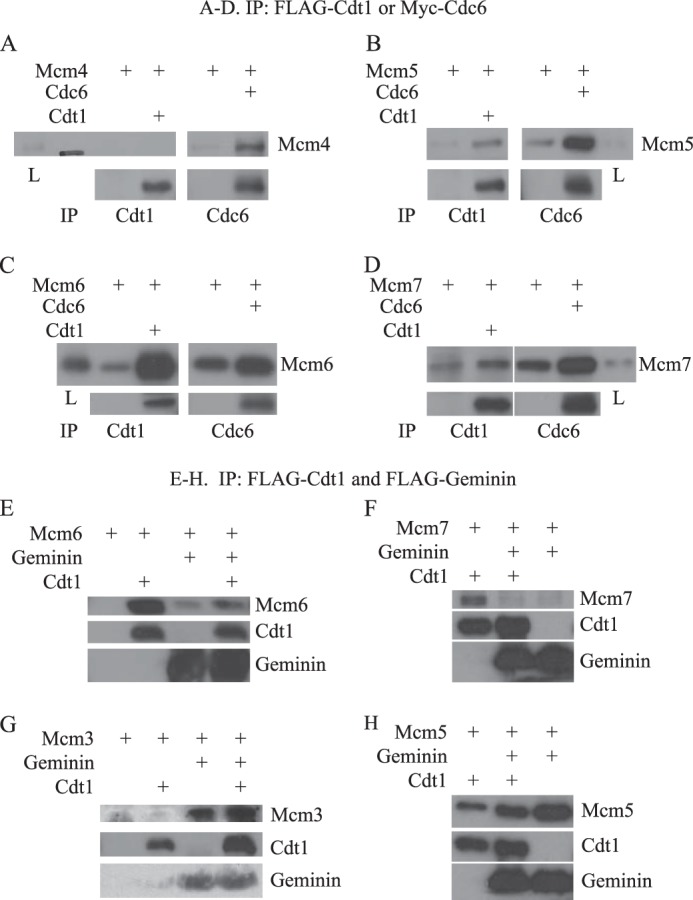
**Interactions between individual human MCM subunits and HsCdt1 or HsCdc6, and effects of HsGeminin on the interactions between MCM subunits and HsCdt1.** *A–D*, extracts of Sf9 cells expressing untagged human Mcm4 (*A*), Mcm5 (*B*), Mcm6 (*C*), or Mcm7 (*D*) were incubated at 4 °C for 3 h with extracts from control cells or extract from cells expressing FLAG-HsCdt1 or Myc-HsCdc6. For each Mcm subunit, an equal amount of protein was added in each reaction. The proteins precipitated by anti-FLAG- or anti-Myc-agarose beads were analyzed by Western blotting. *L* indicates 8.3% of the load. *E–H*, extracts of Sf9 cells expressing untagged human Mcm3 (*G*), Mcm5 (*H*), Mcm6 (*E*), or Mcm7 (*F*) were incubated at 4 °C for 3 h with extracts from control cells or extracts from cells expressing FLAG-HsCdt1 and/or FLAG-HsGeminin-His_6_. The proteins precipitated by anti-FLAG-agarose beads were analyzed by Western blotting. *IP*, immunoprecipitated.

To further explore possible interactions between HsGeminin and the other components of the pre-RC, including HsMCM2–7, we carried out immunoprecipitation experiments with individual HsMCM subunits and HsCdc6, HsCdt1, or HsGeminin. Extracts from Sf9 cells expressing each HsMCM subunit were incubated with extracts of cells expressing epitope-tagged HsCdc6, HsCdt1, or HsGeminin, and the amount of MCM subunit co-precipitated with the tagged proteins was assessed (Fig. 5). We observed the following interactions: HsMcm4, HsMcm5, HsMcm6, and HsMcm7 co-precipitated with HsCdc6, whereas HsMcm5, HsMcm6, and HsMcm7 co-precipitated with HsCdt1 (Fig. 5, *A–D*). These results are consistent with our observations that either HsCdc6 or HsCdt1 can recruit MCM complexes to DNA in the presence of HsORC, albeit with somewhat different efficiencies. We did not observe any association of HsMcm2 or HsMcm3 with either HsCdc6 or HsCdt1 (data not shown). Both HsMcm3 and HsMcm5 co-precipitated with HsGeminin (Fig. 5, *G* and *H*). HsGeminin did not significantly co-precipitate with HsMcm6 or HsMcm7, and inhibited the interaction between these two HsMCM subunits and HsCdt1 (Fig. 5, *E* and *F*). This finding is consistent with our observation that HsGeminin inhibits the recruitment of the HsMCM467 complex to DNA in the presence of HsORC, HsCdc6, and HsCdt1. No interaction between HsGeminin and HsMcm2 or HsMcm4 was detected (data not shown). Therefore, HsGeminin only interacts with the HsMcm3 and HsMcm5 subunits, in agreement with our observation of preferential binding of HsGeminin to HsMCM2–7, but not HsMCM467, in DNA binding experiments (Fig. 4*B*).

Our data provide evidence for mutual interactions among HsCdt1, HsMCM2–7, and HsGeminin, and strongly suggest that these interactions stabilize the ternary complex on origin DNA. This hypothesis is consistent with the finding that the binding sites in Cdt1 for Geminin and MCM2–7 do not overlap (30, 40). Although our data indicate that HsGeminin inhibits the association of HsCdt1 with DNA, in either the presence or absence of HsORC and HsCdc6, it is clear that this inhibitory effect is overcome in DNA-protein complexes containing HsMCM2–7. The interactions that we have described account for the finding that such complexes contain HsGeminin, as well as the pre-RC proteins. Thus, the data suggest that the inhibitory effect of HsGeminin on initiation of DNA replication is not due to blocking assembly of the pre-RC proteins into a complex with DNA, but to interference with some subsequent step in the reaction.

##### HsGeminin Inhibits the Formation of a Salt-stable Complex of HsMCM2–7 with DNA

**FIGURE 6.**
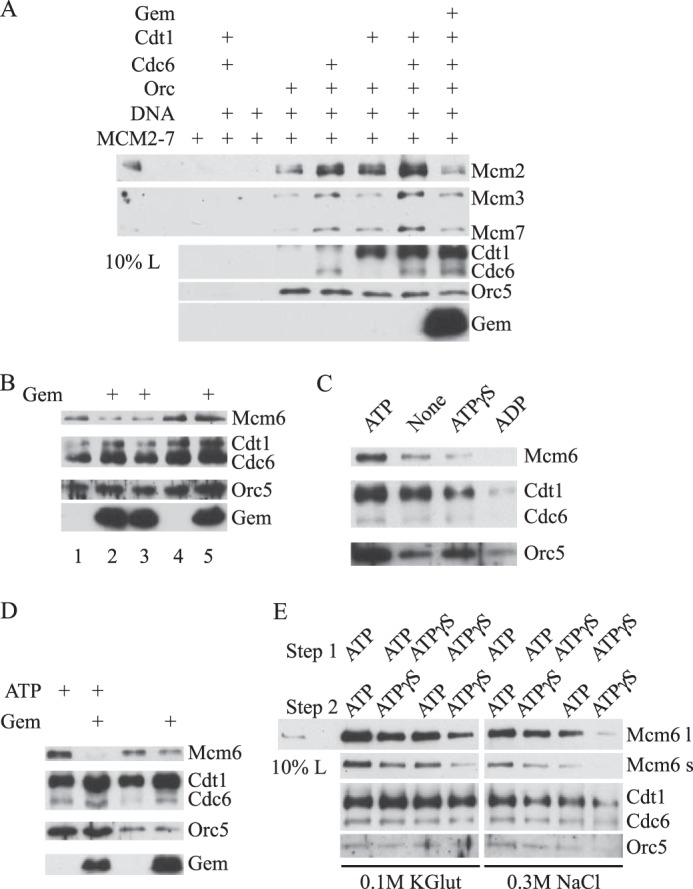
**HsGeminin inhibits the ATP-dependent salt-stable binding of HsMCM2–7 to DNA.** *A*, requirements for formation of salt-stable association of HsMCM2–7 with DNA. Reaction mixtures were assembled and incubated as described in the legend to Fig. 3*B*. The beads were then washed with buffer containing 0.3 m NaCl and bound proteins were analyzed by Western blotting. *B*, HsGeminin inhibits formation, but not the maintenance of salt-stable HsMCM2–7·DNA complexes. Reaction mixtures were assembled and incubated as described in the legend to Fig. 4*C*, except that HsMCM2–7 was used instead of HsMCM467, and HsCdc6 was also present. The beads were then washed with buffer containing 0.5 m NaCl. *Lane 1*: control buffer was added at *t* = −30 min. *Lane 2*: HsGeminin was added at *t* = −30 min. *Lane 3*: HsGeminin was added at *t* = 0 min. *Lane 4*: control buffer was added at *t* = +20 min. *Lane 5*: HsGeminin was added at *t* = +20 min. *C*, ATP-dependent formation of salt-stable HsMCM2–7·DNA complexes. Reactions were performed in the presence of ATP, ATPγS, ADP, or control buffer as indicated. The beads were then washed with buffer containing 0.3 m NaCl. *D*, HsGeminin inhibition of salt-stable complex formation. Reaction mixtures were incubated in the presence or absence of ATP as indicated. HsCdt1 was preincubated with control buffer or HsGeminin. The beads were then washed with buffer containing 0.3 m NaCl. *E*, ATP requirement in two-step DNA binding assays. Magnetic beads containing pUC19-lamin-B2 plasmid DNA were incubated with HsORC for 30 min, and then further incubated with HsMCM2–7, HsCdt1, and HsCdc6 for 30 min. ATP or ATPγS was added in each step as indicated. The beads were then washed with buffer containing 0.1 m potassium glutamate or 0.3 m NaCl and analyzed by Western blotting. *Mcm6 l*, long exposure of Mcm6 signal. *Mcm6 s*, short exposure of Mcm6 signal. *Gem*, Geminin.

Studies in *S. cerevisiae* and *Xenopus* systems have suggested that origin-bound MCM complexes can exist in two forms: “associated” or “loaded” (41, 42). Associated MCM complexes are bound to chromatin via interactions with other pre-RC components and dissociate in the presence of high concentrations of salt, whereas loaded MCM complexes remain bound to chromatin in the absence of other pre-RC proteins and are resistant to treatment with high salt. Because, as described above, HsGeminin does not affect the recruitment of HsMCM2–7 to origin DNA in the presence of HsORC, HsCdc6, and HsCdt1 under standard reaction conditions (100 mm potassium glutamate), we asked whether HsGeminin might prevent the formation of complexes that are stable at higher ionic strength. The experiments were carried out as described in the legend to Fig. 3*B*, except that the DNA-protein complexes were washed with buffer containing 0.3 m NaCl (Fig. 6*A*). As expected, the maximal amount of HsMCM2–7 was recruited to DNA when HsORC, HsCdc6, and HsCdt1 were all present in the reaction, and the resulting complexes were stable to washing at a high salt concentration. However, preincubation of HsCdt1 with ∼16-fold excess of HsGeminin greatly reduced the amount of HsMCM2–7 retained on DNA beads (21 ± 7% of the level of binding without HsGeminin). This result is in contrast to the finding described above, that HsGeminin does not prevent the formation of a complex of the pre-RC proteins that is stable to washing at a low salt concentration (Fig. 6*A versus* 3*B*). We conclude that at least two distinct HsMCM2–7·DNA complexes can be formed following incubation of the pre-RC components with origin DNA: a salt-labile and a salt-stable complex. In the presence of HsGeminin, the salt-labile complex readily forms, but the formation of the salt-stable complex is blocked.

To determine whether HsGeminin affected the formation or the maintenance of the salt-stable complex, we carried out order of addition experiments (Fig. 6*B*). In these experiments we washed the protein-DNA complexes with 0.5 m NaCl, which gave similar results to washing with 0.3 m NaCl (data not shown). HsGeminin was able to inhibit the formation of salt-stable HsMCM2–7·DNA complexes when preincubated with HsCdt1, as shown previously, or when added to the reaction without preincubation at the same time as HsCdt1, HsCdc6, and HsMCM2–7. However, no inhibition was observed when HsGeminin was added to the reaction 20 min after all of the pre-RC components were added, when significant amounts of salt-stable HsMCM2–7·DNA complexes had already been formed (Fig. 6*B*). These results are similar to those obtained in our study of the recruitment of HsMCM467 (Fig. 4*C*), and are consistent with the hypothesis that one role of HsGeminin is to prevent formation of new pre-RCs without blocking the activity of pre-existing pre-RCs. It is also consistent with the previous observation that Geminin is unable to inhibit MCM loading and DNA replication in crude *Xenopus* extracts when it was added after the recruitment of MCM to chromatin (26).

To determine whether formation of the salt-stable HsMCM2–7·DNA complex is dependent on ATP binding and/or hydrolysis, DNA binding assays were carried out in the presence of ATP, ATPγS, ADP, or control buffer lacking nucleotide. We observed that the amount of salt-stable HsMCM2–7 complex was greatly reduced when ATPγS or ADP was used in place of ATP (Fig. 6*C*). When no nucleotide was added, a small amount of HsMCM2–7 remained on DNA after washing at a high salt concentration, but the stability of these residual HsMCM2–7 complexes was not significantly affected by HsGeminin (Fig. 6*D*). Because our standard DNA binding assays are performed in two steps, incubation of HsORC with origin DNA followed by incubation with HsCdc6, HsCdt1, and HsMCM2–7, we asked whether ATP hydrolysis is required in both steps. When ATP was replaced with ATPγS in both steps, the association of HsMCM2–7 with DNA was greatly reduced after washing at low salt concentration and almost completely eliminated after washing at high salt concentration. A more moderate reduction was observed when ATPγS replaced ATP in either the first or the second step of the reaction. Thus, ATP hydrolysis appears to be required in both steps to achieve the maximal formation of HsMCM2–7·DNA complexes.

## DISCUSSION

### 

#### 

##### In Vitro Assembly of the Human Pre-RC Complex

In this study, we have reconstituted complexes of purified human pre-RC proteins and studied the interactions required for efficient pre-RC assembly. Our data indicate that the association of HsORC with DNA significantly enhances the recruitment of both HsCdc6 and HsCdt1. HsCdc6 and HsCdt1 promote the binding of each other to DNA, both in the presence and absence of HsORC. Thus, the assembly of the initial complex containing HsORC, HsCdc6, and HsCdt1 is stabilized by all possible binary interactions among the three components. These interactions are similar to those identified in various studies in *Saccharomyces cerevisiae*, *Schizosaccharomyces pombe*, and *Xenopus* systems using similar approaches (1, 2, 35, 43, 44).

HsORC, HsCdc6, and HsCdt1 are all required for the maximal recruitment of the HsMCM complexes, HsMCM2–7 and HsMCM467. Our data suggest that the requirement for HsORC for initial assembly may be largely an indirect result of its role in recruiting HsCdt1 and HsCdc6. We observed multiple direct interactions between isolated MCM subunits and HsCdc6 and HsCdt1: subunits Mcm4, Mcm5, Mcm6, and Mcm7 interact with HsCdc6, whereas subunits Mcm5, Mcm6, and Mcm7 interact with HsCdt1. In addition, we observed that HsCdc6 and HsCdt1 are sufficient to recruit the MCM complexes in the absence of HsORC. Finally, we observed only weak interactions between HsORC and either HsMCM467 or HsMCM2–7. Thus, our data suggest a model in which mutual interactions among HsORC, HsCdc6, and HsCdt1 stabilize a quaternary complex with DNA that recruits HsMCM complexes largely via interactions with the HsCdc6 and HsCdt1 proteins.

##### HsGeminin Does Not Inhibit the Recruitment of HsMCM2–7 to DNA by HsORC, HsCdc6, and HsCdt1

Several different mechanisms have been proposed to explain how Geminin blocks loading of MCM complexes at origins of DNA replication: 1) inhibition of the interaction between Cdt1 and MCM complexes (30, 32, 39, 45), 2) inhibition of the interaction between Cdt1 and DNA (30), and 3) inhibition of histone acetylation by the acetylase HBO1, which is recruited to origin DNA in G_1_ by Cdt1 (46–48). In agreement with an earlier report (27), we observed that HsGeminin forms a stable complex with HsCdt1, which is resistant to 0.5 m NaCl. This complex has significantly reduced DNA-binding activity relative to free HsCdt1, both in the absence and presence of HsORC and HsCdc6. Because HsCdt1 plays a crucial role in recruiting HsMCM467 to DNA, the decreased association of HsCdt1 with DNA would be expected to reduce HsMCM467 recruitment. However, our results indicate that the recruitment of HsCdt1 was not affected by HsGeminin when HsMCM complexes were present in the reaction. Therefore, the reduced affinity of HsCdt1 for DNA was probably overcome by the interactions between HsCdt1 and other proteins present in the reaction. We also observed that HsGeminin directly interferes with the interaction between HsCdt1 and HsMcm6 and HsMcm7, which is most likely the reason for the reduced recruitment of HsMCM467 in the presence of HsGeminin observed in this study.

Surprisingly, HsGeminin did not inhibit the recruitment of HsMCM2–7 *in vitro*. This finding can be explained by our discovery of a previously unknown interaction between HsGeminin and HsMCM2–7. We showed directly that HsGeminin can be recruited to DNA by HsMCM2–7 in the absence of HsCdt1 and that HsGeminin interacts directly with the HsMcm3 and HsMcm5 subunits. Therefore, quinary complexes of HsORC·HsCdc6·HsCdt1·HsGeminin·HsMCM2–7 are probably formed on DNA. These findings indicate that HsGeminin does not block the initial assembly of pre-RC components at origins of DNA replication and raise the possibility that HsGeminin may affect the activity of the MCM or CMG complexes (see below).

##### HsGeminin Inhibits the Formation of a Salt-stable HsMCM2–7 Complex on DNA

Our data suggest that two distinct HsMCM2–7·DNA complexes can be formed following incubation of the pre-RC components with origin DNA: a salt-labile and a salt-stable complex. This observation is analogous to findings in several studies in *S. cerevisiae* and *Xenopus*, which identified a salt-sensitive MCM-DNA complex whose stability is dependent upon the presence of the other pre-RC components and a salt-resistant MCM-DNA complex, whose stability is independent of the other pre-RC components (41, 42). Recent studies have shown that the *S. cerevisiae* salt-resistant complex consists of the MCM complex encircling double-stranded DNA (1, 2). Our data indicate that HsGeminin has different effects on the formation of the two human complexes, having no effect on the formation of the salt-labile complex, but strongly inhibiting the formation of the salt-stable complex. This observation is similar to previous results obtained using *Xenopus* extracts, which showed that Geminin did not affect the association of MCM complexes with plasmids, but significantly reduced the amount of DNA-bound MCM complexes after Triton treatment (49). Importantly, the formation of salt-stable association of HsMCM2–7 with DNA is dependent on ATP hydrolysis. Studies in *S. cerevisiae* indicated that ATP hydrolysis by Cdc6 is required for the initial formation of salt-resistant (loaded) MCM2–7 complexes (50), whereas ATP hydrolysis by ORC is required for reiterative MCM2–7 loading (9). In our study, pre-RC assembly *in vitro* was performed in two steps. In the first step origin DNA was incubated with HsORC, and in the second step pre-formed ORC-DNA complexes were incubated with HsCdc6, HsCdt1, and HsMCM2–7. Because we observed that ATP hydrolysis was required in both steps for maximum formation of salt-stable complexes, it seems likely that efficient loading of HsMCM2–7 on the DNA requires the ATPase activities of both HsCdc6 and HsORC.

The kinetic relationship of the salt-sensitive and salt-stable MCM-DNA complexes was not defined by our studies, but one reasonable possibility is that the salt-sensitive complex is a precursor to the salt-stable complex. If this is the case, our data would suggest that HsGeminin inhibits the transition from an initial complex dependent on protein-protein interactions among HsORC, HsCdc6, HsCdt1, and HsMCM2–7 to a more stable complex that is competent for initiation of DNA replication, perhaps analogous to the yeast complex containing MCM proteins topologically bound to DNA. It is quite possible that the negative effects of HsGeminin on the interactions between HsCdt1 and DNA, and between HsCdt1 and HsMcm6 and HsMcm7, play important roles in this inhibition, although these effects were overcome at earlier stages of pre-RC formation.

##### The Interaction between HsGeminin and HsMCM2–7 Complex

As noted above, it has been suggested that Geminin functions to inhibit initiation of DNA replication by perturbing the interaction of Cdt1 with DNA, MCM complexes, or chromatin factors. Our observation that HsGeminin interacts with HsMCM2–7 raises the possibility that HsGeminin might have additional functions, such as directly modifying the activity of the MCM-dependent helicase. Chromatin immunoprecipitation experiments in HeLa cell extracts revealed colocalization of HsGeminin and HsMcm7 at regions bracketing HsORC binding sites in early S phase, when the level of HsCdt1 is low, suggesting that the interaction between HsGeminin and HsMCM2–7 is maintained after assembly of the pre-RC (51). The localization of HsGeminin does not change as S phase progresses, indicating that CMG complexes engaged in active DNA chain elongation do not contain associated HsGeminin. We speculate that HsGeminin is able to bind to HsMCM2–7 after the latter has been loaded onto origin DNA, and helps to maintain the associated MCM2–7 in an inactive state until the onset of DNA chain elongation. HsMcm3 and HsMcm5, the two MCM subunits that interact with HsGeminin *in vitro*, are both considered regulatory subunits of the MCM2–7 complex. It has been suggested that a conformational change in Mcm2 and Mcm5 may regulate the helicase activity of *S. cerevisiae* MCM2–7 (52). Recent work has also suggested that ATPase active sites at the interfaces of MCM subunits Mcm6/2 and Mcm5/3 modulate the biochemical activities of Mcm2 and Mcm5 (53). It is possible that binding of HsGeminin to HsMcm3 and HsMcm5 prevents the conformational change required for helicase loading or activity. It has also been observed that *S. cerevisiae* Mcm5 is involved in a Cdc7-activated molecular switch that is required for initiation of DNA replication (54). The requirement for Cdc7 can be bypassed by a point mutation (P83L) in Mcm5, and the equivalent mutation in the archaeon *Methanobacterium thermoautotrophicum* MCM protein induces a significant conformational change, suggesting a structural basis for the Cdc7-mediated switch (55). Thus, it is also possible that the interaction between HsGeminin and HsMcm5 inhibits initiation of DNA replication by blocking this conformational change. In any case, it is clear that HsGeminin blocks a crucial step(s) in the initiation reaction after association of the pre-RC proteins.

## Footnotes

6 The abbreviations used are:

ORC: origin recognition complex
pre-RCs: pre-replicative complexes
MCM: mini-chromosome maintenance
Hs: *Homo sapiens*
CMG: Cdc45-MCM2–7-GINS
Ni-NTA: nickel-nitrilotriacetic acid
ATPγS: adenosine 5′-*O*-(thiotriphosphate).
